# HPN, a Synthetic Analogue of Bromophenol from Red Alga *Rhodomela confervoides*: Synthesis and Anti-Diabetic Effects in C57BL/KsJ-*db*/*db* Mice

**DOI:** 10.3390/md11020350

**Published:** 2013-01-30

**Authors:** Dayong Shi, Shuju Guo, Bo Jiang, Chao Guo, Tao Wang, Luyong Zhang, Jingya Li

**Affiliations:** 1 Institute of Oceanology, Chinese Academy of Sciences, Qingdao 266071, China; E-Mails: guoshuju@qdio.ac.cn (S.G.); beckyjiang0220@163.com (B.J.); yujuejie0546@126.com (C.G.); 2 Nantong Branch, Institute of Oceanology, Chinese Academy of Sciences, Nantong 226006, China; 3 Jiangsu Center for Drug Screening, China Pharmaceutical University, Nanjing 210009, China; E-Mails: wangtao1331@126.com (T.W.); lyzhang@cpu.edu.cn (L.Z.); 4 National Center for Drug Screening, Shanghai Institute of Materia Medica, Shanghai Institutes for Biological Sciences, Chinese Academy of Sciences, Shanghai 201203, China; E-Mail: jyli@mail.shcnc.ac.cn

**Keywords:** bromophenol, protein tyrosine phosphatase 1B inhibitor, *db*/*db* mouse model, anti-diabetes properties

## Abstract

3,4-dibromo-5-(2-bromo-3,4-dihydroxy-6-(isopropoxymethyl)benzyl)benzene-1,2-diol (HPN) is a synthetic analogue of 3,4-dibromo-5-(2-bromo-3,4-dihydroxy-6-(ethoxymethyl)benzyl)benzene-1,2-diol (BPN), which is isolated from marine red alga *Rhodomela confervoides* with potent protein tyrosine phosphatase 1B (PTP1B) inhibition (IC_50_ = 0.84 μmol/L). The *in vitro* assay showed that HPN exhibited enhanced inhibitory activity against PTP1B with IC_50_ 0.63 μmol/L and high selectivity against other PTPs (T cell protein tyrosine phosphatase (TCPTP), leucocyte antigen-related tyrosine phosphatase (LAR), Src homology 2-containing protein tyrosine phosphatase-1 (SHP-1) and SHP-2). The results of antihyperglycemic activity using *db*/*db* mouse model demonstrated that HPN significantly decreased plasma glucose (*P* < 0.01) after eight weeks treatment period. HPN lowered serum triglycerides and total cholesterol concentration in a dose-dependent manner. Besides, both of the high and medium dose groups of HPN remarkably decreased HbA1c levels (*P* < 0.05). HPN in the high dose group markedly lowered the insulin level compared to the model group (*P* < 0.05), whereas the effects were less potent than the positive drug rosiglitazone. Western blotting results showed that HPN decreased PTP1B levels in pancreatic tissue. Last but not least, the results of an intraperitoneal glucose tolerance test in Sprague–Dawley rats indicate that HPN have a similar antihyperglycemic activity as rosiglitazone. HPN therefore have potential for development as treatments for Type 2 diabetes.

## 1. Introduction

Type 2 diabetes mellitus (T2DM) is a complex endocrine and metabolic disorder. On the World Diabetes Day 2011, the International Diabetes Federation’s 5th edition of the Diabetes Atlas indicated that the number of people living with diabetes was expected to rise from 366 million in 2011 to 552 million by 2030, if no urgent action is taken. Furthermore, the disease is now being observed with increasing frequency in young children and adolescents. Current treatment agents for T2DM mainly include insulin, biguanides, sulfonylureas, α-glucosidase inhibitors and thiazolidinediones (TZDs). However, adverse effects such as hypoglycemia, weight gain and edema limit their clinical use [1].

Protein tyrosine phosphatase 1B (PTP1B), the first characterized protein tyrosine phosphatase (PTPases), has become a new target for the treatment of T2DM because of its involvement in the insulin signaling cascade as a major negative regulator [2]. In addition, molecular biology investigations have already proven that PTP1B knock-out mice exhibit the phenotypes of increased insulin sensitivity, improved glucose tolerance, and resistance to diet-induced obesity [3,4]. Based on the above research results, the inhibition of PTP1B has emerged as a novel therapeutic strategy for the treatments of Type 2 diabetes mellitus.

In the past decades, there have been a number of reports about the development of synthetic PTP1B inhibitors [5,6,7]. However, the highly charged phosphatase active site and the relatively shallow nature of the surrounding protein surface pose a great challenge to the discovery of cell permeable and orally bio-available PTP1B inhibitors. At the same time, more and more studies have been focusing on PTP1B inhibitors isolated from plants, and most of them have demonstrated promising results [8,9,10,11,12,13,14,15,16,17,18,19].

**Figure 1:**
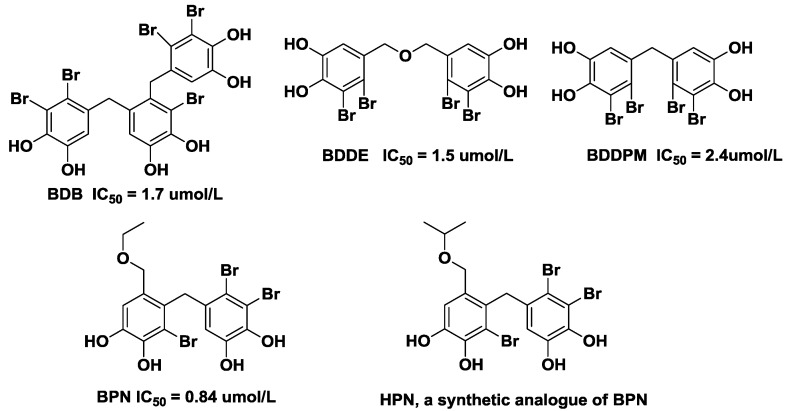
Structures of 3,4-dibromo-5-(2-bromo-3,4-dihydroxy-6-(isopropoxymethyl)benzyl)benzene-1,2-diol (HPN) and four bromophenols isolated from red alga *Rhodomela confervoides*.

Bromophenols exists in most marine organisms, such as mollusks, algae, jellyfish and sponges. Accumulated studies have reported that bromophenols exhibit a wide spectrum of biological and pharmacological activities including antioxidant activity [20,21], α-glucosidase inhibition [22], and antitumor effects [23]. Four novel bromophenols from the ethanolic extract of the red alga *Rhodomela confervoides* were isolated and screened as PTP1B inhibitors in our lab (Figure 1) [24,25]. In order to obtain new types of PTP1B inhibitors, a series of bromophenol derivatives were synthesized by using bis-(2,3-dibromo-4,5-dihydroxy-phenyl)-methane (BDDPM) and 3-bromo-4,5-bis(2,3-dibromo-4,5-dihydroxybenzyl)-1,2-benzenediol (BDB) as lead compounds. Fortunately, two of the bromophenol derivatives exhibited enhanced PTP1B inhibitory activity and are promising development prospects [26,27]. In the present study, we selected BPN (3,4-dibromo-5-(2-bromo-3,4-dihydroxy-6-(ethoxymethyl)benzyl)benzene-1,2-diol) as the lead compound due to its potent PTP1B inhibition (IC_50_ = 0.84 μmol/L). Optimization of BPN provided a novel bromophenol analogue HPN (3,4-dibromo-5-(2-bromo-3,4-dihydroxy-6-(isopropoxymethyl)benzyl)benzene-1,2-diol) and its structure is in Figure 1. Herein, we report the synthesis of HPN and use the diabetic mouse strain C57BL/KsJ-db/db (*db*/*db*), which exhibits many of the metabolic disturbances of human Type 2 diabetes including hyperglycemia, obesity, and early hyperinsulinemia to investigate the pharmacological properties of HPN.

## 2. Results and Discussion

### 2.1. Characterization and Identification of HPN

HPN was obtained as a yellowish powder. The electron ionization mass spectroscopy (EIMS) spectrum showed the tribrominated molecular ion peak cluster at *m*/*z* 544/542/540/538 (1:3:3:1). The molecular formula was determined as C_17_H_16_Br_3_O_5_ by HREIMS at *m*/*z* 536.8525 (calcd for C_17_H_16_Br_3_O_5_ 536.8548). The ^1^H NMR spectrum revealed the presence of four phenol hydroxyl protons (δ 9.72, δ 9.69, δ 9.26, δ 9.11), two phenyl ring protons (δ 6.88, δ 6.05), two methylene protons (δ 4.15, δ 3.98) and the isopropyl group (δ 3.51, δ 1.02). The ^13^C NMR spectrum showed the signals representing twelve phenyl ring carbons (δ 144.9–113.0), one methine carbon (δ 79.1, δ 67.6, δ 38.3) and two methyl carbons (δ 21.7). Detailed spectrum data are: ^1^H-NMR (500 MHz, DMSO-*d*_6_) δ: 9.72 (s, 1H, OH), 9.69 (s, 1H, OH), 9.26 (s, 1H, OH), 9.11 (s, 1H, OH), 6.88 (s, 1H), 6.05 (s, 1H), 4.15 (s, 2H), 3.98 (s, 2H), 3.51 (m, *J* = 6.43 Hz, 2H), 1.02 (d, *J* = 6.43 Hz, 6H); ^13^C-NMR (125 MHz, DMSO-*d*_6_) δ: 144.9 (C), 144.3 (C), 142.5 (C), 142.4 (C) 130.5 (C), 129.3 (C), 127.5 (C), 115.4 (CH), 114.5 (C), 114.3 (C), 113.9 (CH), 112.9 (C), 79.1 (CH), 67.6 (CH_2_) 38.3 (CH_2_), 21.7 (2 × CH_3_). EIMS *m*/*z* (% relative intensity): 544/542/540/538 [M]^+^ (1/3/3/1), 484/482/480/478 (2/7/7/2), 467/465/463/461 (2/6/6/2), 403/401/399 (5/10/5), 322/320 (10/9), 82/80 (98/100), 59 (30). HRMS *m*/*z* calcd for C_17_H_16_O_5_Br_3_ [M − H]^−^, 536.8548; found, 536.8525.

### 2.2. Enzyme-Based Inhibitory Activities Assays *in Vitro*

We firstly evaluated the inhibition of HPN against recombinant PTP1B *in vitro*. HPN exhibited remarkable inhibitory activity against PTP1B with IC_50_ 0.63 μmol/L, which was more potent than BPN (IC_50_ = 0.84 μmol/L). Selectivity is one of the major issues for the development of PTP1B inhibitors as drugs, hence, we investigated the selectivity of HPN against other PTPs (TCPTP, LAR, SHP-1 and SHP-2), which share a high degree of structural conservation in the active site. We found that HPN demonstrated an excellent selectivity against TCPTP, LAR, SHP-1 and SHP-2 (>50-fold).

### 2.3. Effects of HPN on Body Weight, Food and Water Intake

The typical symptoms of diabetes are polydipsia, polyuria, and polyphagia, so we studied the effects on body weight, food and water intake after HPN administration for 8 weeks in *db*/*db* mice. Since no small molecule inhibitors of PTP1B are on the market, we selected rosiglitazone as the positive control, which is an effective antidiabetic drug and works as an insulin sensitizer. During the 8-week period, there were no significant changes in defecation and urination of mice treated with HPN and rosiglitazone, and no mortality occurred. As shown in Table 1, the initial and final body weights of the *db*/*db* mice were significantly higher than those in *db*/*dm* mice. There was no significant difference in the food and water intake between the model and control groups. The body weights increased gradually during the treatment period. At the end of the study, the mice in the rosiglitazone treated group had a significantly higher body weight compared to the HPN treated groups. The mice in the HPN treated group exhibited greater body weight gain, however, the increase was less than that observed in the rosiglitazone treated group. In the low-dose treated group, the body weight was obviously lower than in the model group (*P* < 0.05). HPN at different doses reduced the food and water intake in *db*/*db* mice, but the differences were not significant. The results indicated that HPN could reduce diabetes-related symptoms.

**Table 1:** Effects of HPN on food and water intake, and body weight in *db*/*db* mice.

Group	Body weight (g) ^a^		Food intake (g/day) ^a^		Water intake (mL/day) ^a^	
	initial	final	initial	final	initial	final
Control	17.7 ± 0.9	22.0 ± 2.5	3.42 ± 0.03	3.26 ± 0.40	3.46 ± 0.04	3.67 ± 0.19
Model	36.6 ± 2.1 **	53.7 ± 2.6 **	5.05 ± 0.03	7.67 ± 0.62	7.55 ± 0.17	13.12 ± 0.95
HPN (40 mg/kg)	35.9 ± 1.6	52.3 ± 3.1	4.88 ± 0.07	5.46 ± 0.89	7.89 ± 0.13	9.94 ± 0.41
HPN (20 mg/kg)	35.7 ± 1.8	50.5 ± 4.4	4.85 ± 0.02	6.54 ± 0.70	7.96 ± 0.34	12.00 ± 0.39
HPN (10 mg/kg)	35.7 ± 1.5	48.1 ± 3.0 ^#^	4.79 ± 0.08	6.65 ± 0.68	7.67 ± 0.04	12.92 ± 0.36
Rosiglitazone (20 mg/kg)	36.0 ± 3.1	54.8 ± 4.1	5.03 ± 0.06	7.09 ± 0.48	8.41 ± 0.49	11.72 ± 0.34

^a^ Data are expressed as means ± SD, *n* = 10; ^#^ *P* < 0.05; ** *P* < 0.01 when compared to the control group.

### 2.4. Effects of HPN on Blood Glucose

**Figure 2:**
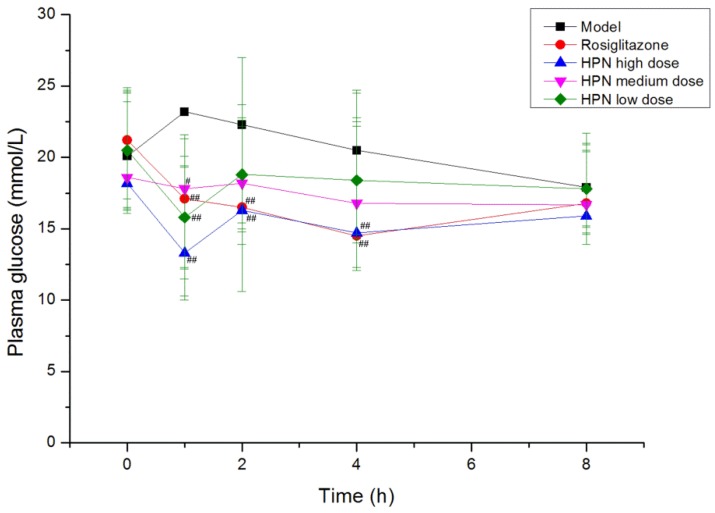
The time-effect relationship of HPN on the fifth day in *db*/*db* mice model. ^#^ *P* <0.05, ^##^ *P* <0.01 *vs.* model.

Hyperglycemia plays an important role in the development of Type 2 diabetes mellitus, therefore, the effective control of the blood glucose level is crucial for preventing diabetic complications and improving the quality of the life in diabetic patients. Firstly, we examined the time-effect relationship of the compound in a *db*/*db* mice model on the fifth day. As shown in Figure 2, the high dose of HPN produced a remarkable reduction in plasma glucose at 1 h, 2 h and 4 h during the intervention period (*P* < 0.01, *P* < 0.05), and its greatest effect was 1 h after intragastric administration, lowering plasma glucose from 18.2 mmol/L to 13.3 mmol/L (*P* < 0.01). At that time, a notable reduction was observed in the medium and low dose groups of HPN (*P* < 0.01, *P* < 0.05). There was no decline of effect in plasma glucose prolonged to 8 h and the plasma glucose maintained at 16 mmol/L. The antihyperglycemic activity results of HPN are illustrated in Table 2, the positive control rosiglitazone decreased plasma glucose levels from 18.7 mmol/L to 15.5 mmol/L in the first week (*P* < 0.05). Similarly, HPN at 40 mg/kg significantly decreased plasma glucose (*P* < 0.01) and maintained the plasma glucose level at 15 mmol/L after an 8-week treatment period. In general, the high dose and medium dose of HPN were more effective in decreasing the blood glucose level compared to the low dose group of HPN.

**Table 2:** Effects of HPN on blood glucose level in *db*/*db* mice.

Group	Dose (mg/kg)	Plasma glucose (mmol/L) ^a^			
		Baseline	1 week	4 weeks	8 weeks
Control		6.0 ± 0.6	6.0 ± 0.4	6.1 ± 0.3	5.8 ± 0.4
Model		18.9 ± 4.5 **	20.0 ± 3.6 **	20.2 ± 4.6 **	18.9 ± 4.1 **
HPN	40	19.8 ± 5.0	15.1 ± 3.6 ^##^	14.7 ± 3.7 ^##^	15.8 ± 2.4 ^#^
	20	18.6 ± 4.5	16.6 ± 3.4 ^#^	15.4 ± 4.7 ^#^	15.5 ± 3.3 ^#^
	10	19.4 ± 4.9	16.0 ± 3.7 ^#^	17.5 ± 3.9	16.5 ± 3.5
Rosiglitazone	20	18.7 ± 3.8	15.5 ± 4.0 ^#^	15.6 ± 3.1 ^#^	13.9 ± 2.9 ^##^

^a^ Data are expressed as means ± SD, *n* = 10; ^#^ *P* < 0.05, ^##^ *P* < 0.01 when compared to the model group; ** *P* < 0.01 when compared to the control group.

### 2.5. Effects of HPN on Serum Lipids

It is well documented that there is an elevation of serum lipid concentration in diabetics. As illustrated in Table 3, the *db*/*db* mice used in this study had significantly elevated triglyceride and total cholesterol concentrations compared to the *db*/*dm* mice (*P* < 0.01, *P* < 0.05). The serum triglyceride and total cholesterol levels of the rosiglitazone treated group were much lower than in the model group (*P* < 0.01, *P* < 0.05). The HPN lowered the triglycerides and total cholesterol concentration in a dose-dependent manner in the HPN-treated group, and the high-dose group showed a better lipid lowering profile (*P* < 0.05) than other dose groups. 

**Table 3:** Effects of HPN on serum lipid in *db*/*db* mice.

Group	Dose (mg/kg)	Triglycerides (mmol/L) ^a^				Total cholesterol (mmol/L) ^a^		
		Baseline	4 weeks	8 weeks	Baseline		4 weeks	8 weeks
Control		1.19 ± 0.25	1.30 ± 0.26	1.19 ± 0.24	3.01 ± 0.83		3.32 ± 0.80	2.99 ± 0.72
Model		1.47 ± 0.30 *	1.69 ± 0.39 *	1.74 ± 0.40 **	4.20 ± 1.17 *		7.22 ± 2.91 **	7.18 ± 2.18 **
HPN	40	1.36 ± 0.35	1.32 ± 0.21 ^#^	1.32 ± 0.34 ^#^	3.89 ± 0.93		4.67 ± 1.55 ^#^	5.01 ± 1.64 ^#^
	20	1.44 ± 0.42	1.32 ± 0.27 ^#^	1.36 ± 0.31 ^#^	3.86 ± 1.42		5.01 ± 1.45	5.12 ± 2.31
	10	1.44 ± 0.28	1.45 ± 0.30	1.47 ± 0.32	4.72 ± 0.91		5.54 ± 1.59	5.79 ± 1.69
Rosiglitazone	20	1.46 ± 0.23	1.33 ± 0.27 ^#^	1.36 ± 0.23 ^#^	4.19 ± 1.04		4.67 ± 1.42 ^#^	4.44 ± 1.60 ^##^

^a^ Data are expressed as means ± SD, *n* = 10; ^#^ *P* < 0.05, ^##^ *P* < 0.01 when compared to the model group; ** *P* *<* 0.01 when compared to the control group.

### 2.6. Effects of HPN on Glycated Hemoglobin and Glycated Serum Protein

Glycated hemoglobin (HbA1c) and glycated serum proteins (GSP) are well known parameters of glycemic control. Hence, we were interested to investigate the effect of HPN on HbA1c and GSP levels. As depicted in Table 4, the blood HbA1c and GSP concentrations in the rosiglitazone group were signiﬁcantly lower than in the model group (*P* < 0.01). Also, HPN of high and medium dose groups remarkably decreased HbA1c levels (*P* < 0.05). On the other hand, all dose groups of HPN reduced GSP levels in a dose-dependent manner.

**Table 4:** Effects of HPN on glycated hemoglobin and glycated serum protein.

Group	Dose (mg/kg)	HbA1c ^a^ (%)	GSP ^a^ (mmol/L)
Control		1.1 ± 0.5	1.3 ± 0.2
Model		4.5 ± 1.3 **	4.8 ± 1.0 **
HPN	40	3.2 ± 0.8 ^#^	3.2 ± 0.9 ^##^
	20	3.5 ± 0.9 ^#^	3.3 ± 1.4 ^#^
	10	3.7 ± 1.2	3.7 ± 1.3 ^#^
Rosiglitazone	20	2.8 ± 0.9 ^##^	3.1 ± 1.0 ^##^

^a^ Data are expressed as means ± SD, *n* = 10; ^#^ *P* < 0.05, ^##^ *P* < 0.01 when compared to the model group; ** *P* < 0.01 when compared to the control group.

### 2.7. Effects of HPN on Plasma Insulin Level

In general, the *db*/*db* mice exhibit an initial phase of hyperinsulinemia. In this study, the plasma insulin levels in *db*/*db* mice were significantly higher than in the control group at 4 weeks and 8 weeks (*P* < 0.01). As shown in Table 5, the rosiglitazone treated group had a significantly lower insulin level at 20 mg/kg (*P* < 0.01). As for HPN treated group, the effects were less marked than for the rosiglitazone group. The insulin levels increased during the treatment period from 4 weeks to 8 weeks. Only the high dose of HPN markedly lowered plasma insulin levels compared to the model group (*P* < 0.05). 

**Table 5:** Effects of HPN on plasma insulin level in *db*/*db* mice.

Group	Dose (mg/kg)	Insulin (ng/mL) ^a^	
		4 weeks	8 weeks
Control		1.18 ± 0.37	1.36 ± 0.42
Model		22.56 ± 6.61 **	27.94 ± 4.78 **
	40	15.30 ± 6.26 ^#^	20.80 ± 8.31 ^#^
HPN	20	15.94 ± 7.02	22.22 ± 6.10 ^#^
	10	15.55 ± 3.79 ^#^	23.40 ± 6.50
Rosiglitazone	20	10.60 ± 3.71 ^##^	10.80 ± 3.43 ^##^

^a^ Data are expressed as means ± SD, *n* = 10; ^#^ *P* < 0.05, ^##^ *P* < 0.01 when compared to the model group; ** *P* < 0.01 when compared to the control group.

### 2.8. PTP1B Expression in Pancreatic Tissue (Western Blotting)

**Figure 3:**
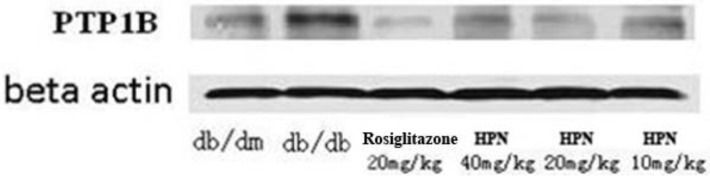
Western blotting assay of HPN.

A Western blotting assay was conducted to verify the expression of PTP1B protein in pancreatic tissue and to determine the inhibition effect of HPN against PTP1B. As illustrated in Figure 3, PTP1B expression in pancreatic tissue of *db*/*db* mice is much higher than in that of *db*/*dm* mice. It is apparent that HPN decreases PTP1B levels at high, medium and low doses, whereas the effects are not as potent as with rosiglitazone.

### 2.9. Intraperitoneal Glucose Tolerance Test in Sprague–Dawley Rats

**Figure 4:**
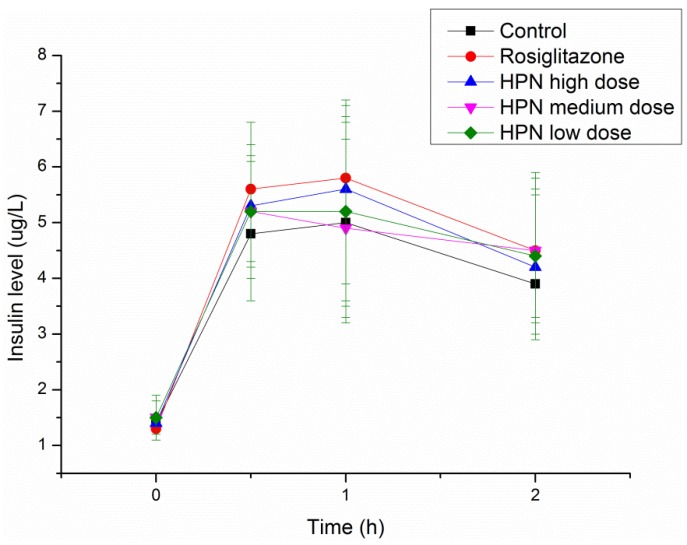
Effects of HPN on insulin level in IPGTT in SD rats.

An intraperitoneal glucose tolerance test (IPGTT) in Sprague–Dawley (SD) rats was performed to investigate the effects of HPN on glucose tolerance during the treatment period and explore the possible mechanism of antihyperglycemic activity of HPN. As illustrated in Table 6, there are no differences of plasma glucose levels among the control, rosiglitazone and HPN groups prior to glucose load. After glucose administration, the plasma glucose levels significantly elevated at 0.5 and 1 h (from 4 mmol/L to 10.7 mmol/L) and decreased to 7.3 mmol/L at 2 h. Similar trends were observed in the rosiglitazone and HPN groups. Furthermore, we examined the insulin levels. As shown in Figure 4, insulin levels in the rosiglitazone and HPN groups were higher than in the control group, but the difference was not statistically significant. In general, as the findings of plasma glucose and insulin levels in the HPN group are similar to those of the rosiglitazone group, it is then implied that HPN conducts its antihyperglycemic activity by enhancing insulin sensitivity. 

**Table 6:** Effects of HPN on blood glucose in intraperitoneal glucose tolerance test (IPGTT) in Sprague–Dawley (SD) rats.

Group	Dose (mg/kg)	Plasma glucose (mmol/L) ^a^			
		0 h	0.5 h	1 h	2 h
Control		4.0 ± 0.4	10.7 ± 3.5	10.7 ± 2.8	7.3 ± 1.4
HPN	40	4.0 ± 0.8	10.4 ± 2.2	10.3 ± 2.1	7.4 ± 0.9
	20	4.0 ± 0.4	10.7 ± 3.1	10.3 ± 2.1	7.8 ± 1.0
	10	4.2 ± 0.5	10.9 ± 2.9	10.8 ± 2.7	8.3 ± 1.1
Rosiglitazone	20	3.9 ± 0.3	9.7 ± 1.7	10.4 ± 2.5	7.4 ± 1.1

^a^ Data are expressed as means ± SD, *n* = 10.

## 3. Experimental Section

### 3.1. General Experimental Procedures

Reagents and all solvents were analytically pure and were used without further purification. All of the experiments were monitored by analytical thin-layer chromatography (TLC) performed on silica gel GF254 precoated plates. After elution, the plate was visualized under UV illumination at 254 nm for UV active materials or coloration by 95% FeCl_3_-EtOH solution. Column chromatography was carried out using silica gel (200–300 mesh). All final compounds were purified to >95% purity, as determined by high-performance liquid chromatography (HPLC). ^1^H-NMR and ^13^C-NMR spectra were recorded on an Inova (500 MHz) NMR spectrometer for proton and at 125 MHz for carbon. Mass spectra and high-resolution mass spectral (HRMS) data were recorded on Autospec Ultima-Tof and Thermo LTQ-Orbitrap mass spectrometer, respectively.

### 3.2. Preparation of HPN

**Scheme 1:**
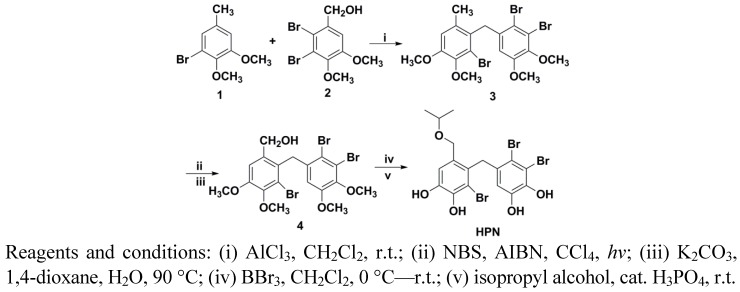
Synthetic route of HPN.

The synthetic route of HPN is shown in Scheme 1. The starting Materials **1** and **2** were prepared according to our previous study [28]. Friedel–Crafts alkylation between **1** and **2** in the presence of AlCl_3_ gave diaryl-methane **3**. Treatment **3** with NBS under *hv* condition provided the benzyl bromide, which was subsequently hydrolysed in the presence of K_2_CO_3_ and 1,4-dioxane gave benzyl alcohol **5**. Demethylation of Compounds **3** and **5** with BBr_3_ in CH_2_Cl_2_ cleanly gave bromophenol in good yields. The bromophenol was treated with isopropyl alcohol in the presence of catalyst H_3_PO_4_ afforded HPN.

### 3.3. Enzymatic Activity Assay *in Vitro*

Recombinant human GST-PTP1B (hGST-PTP1B) protein was used to measure the inhibition of HPN on PTP1B activity, which was determined by monitoring the hydrolysis of pNPP (*para*-nitrophenyl phosphate). Dephosphorylation of pNPP generates the product pNP, which can be monitored at 405 nm. HPN was solubilized in dimethyl sulfoxide (DMSO) and distributed to a 96-well clear polystyrene plate. DMSO was distributed as the full enzyme activity. After adding an assay mixture, GST-PTP1B was added to initiate the reaction. The high-throughput screening was carried out in a mixture containing MOPS, *p*NPP, PTP1B and DMSO, and the catalysis of *p*NPP was continuously monitored at 405 nm for 2 min at 30 °C. The inhibitory rate was calculated according to the formula:

% inhibition = 100 × (*V*_DMSO_ − *V*_sample_)/*V*_DMSO_ (1)

All the assays were performed in triplicate.

IC_50_ value was calculated using GraphPad Prism 4.00 (GraphPad Software Inc., USA). To study the inhibition selectivity on other PTP family members, TCPTP (T-cell protein tyrosine phosphatase), SHP-1 (Src homology 2-containing protein tyrosine phosphatase-1), SHP-2 (Src homology 2-containing protein tyrosine phosphatase-2) and LAR (leucocyte antigen-related tyrosine phosphatase) were prepared as described previously [29]. Assays for these PTPs were performed at the optimal pH for each individual enzyme activity. These enzymes and HPN were pre-incubated for 3 min at 4 °C, and the assays were initiated by adding substrates. Assays performed for SHP-1, SHP-2 and LAR were done using 3-*O*-methylfluorescein phosphate (OMFP) as a substrate.

### 3.4. Animals

C57BL/KsJ *db*/*db* mice (6–8 weeks of age) and their non-diabetic controls (*db*/*dm* mice, 6–8 weeks of age) were purchased from Experimental Animal Center of Military Medical Sciences (Beijing, China). The Sprague–Dawley (SD) rats (180–220 g) were provided by Shanghai SIPPR/BK Lab Animal Ltd. All the animals were left to acclimatize for 1 week before the experimental period. The animals were housed in a room controlled for temperature (24 ± 2.0 °C), relative humidity (60%–80%), and 12/12 h light/dark cycle (lights on at 6:00 am). All the animals were allowed free access to fresh water and laboratory chow.

### 3.5. Experiment Design

The *db*/*db* mice were randomly divided into five groups matched for blood glucose levels, each group consisting of ten mice, as follows:

Model group: *db*/*db* mice given 0.5% CMC-Na and 2% Tween-80.
Positive drug group: *db*/*db* mice given rosiglitazone (20 mg/kg).
HPN high-dose group: *db*/*db* mice given HPN (40 mg/kg).
HPN medium-dose group: *db*/*db* mice given HPN (20 mg/kg).
HPN low-dose group: *db*/*db* mice given HPN (10 mg/kg).

The *db*/*dm* mice were used as control group. Rosiglitazone was dissolved in distilled water and HPN were suspended in 0.5% CMC-Na containing 2% Tween-80 and administered orally for eight weeks (approximately 0.2 mL/20 g body weight). Body weights and food intake were measured weekly. Blood glucose concentrations were checked with ONE TOUCH Ultra^®^ glucometer (LifeScan, USA) weekly. Serum triglyceride and total cholesterol levels were measured by an enzymatic method (Whitman Biotech Co., Ltd., Nanjing, China) every two weeks. At the end of the 8-week treatment period, the mice were fasted overnight. One hour after administration, the mice’s eyeballs were excised for blood sampling. Blood samples were collected into heparin-coated tubes. HbA1c concentration was measured by HbA1c reagent (Whitman Biotech Co., Ltd., Nanjing, China). After centrifugation at 3500× *g* for 15 min at 4 °C, the plasma was carefully removed from the sample. The GSP levels were determined by fructosamine method using the GSP assay kit (Whitman Biotech Co., Ltd., Nanjing, China). The plasma insulin levels were determined using an ELISA kit (ExCell Biology Inc., Shanghai, China).

### 3.6. Western Blotting Assay

Western blotting was performed for the analysis of PTP1B expression in pancreatic tissue using standard protocols. Fourty micrograms of protein were subjected to 10% (w/v) SDS-polyacrylamide gels and transferred to a nitrocellulose membrane. The membrane was blocked for 1 h in tris-buffered saline with tween (TBST) (PBS, 0.05% Tween-20) containing 3% nonfat dry milk and incubated with antibodies for PTP1B (Santa Cruz Biochemicals, Dallas, FL, USA). After they were washed in TBST, the corresponding secondary antibodies were incubated at room temperature for 1 h. The antibody-reactive bands were revealed by the enhanced chemiluminescence kit (Amersham, UK) and were exposed using ChemiDoc XRS Gel Imager (Bio-rad, USA).

### 3.7. Intraperitoneal Glucose Tolerance Test (IPGTT) in SD Rats

The rats were divided into five groups, each group consisting of ten rats, as follows:

Control group: SD rats given 0.5% CMC-Na.
Positive drug group: SD rats given rosiglitazone (20 mg/kg).
HPN high-dose group: SD rats given HPN (40 mg/kg).
HPN medium-dose group: SD rats given HPN (20 mg/kg).
HPN low-dose group: SD rats given HPN (10 mg/kg).

The IPGTT was conducted at the end of 7-day treatment. SD rats were fasted overnight (16–18 h) and then given vehicle, positive drugs and HPN as mentioned above. Five hours after dosing, the fasting blood glucose level was measured from tail-tip blood using the ONE TOUCH Ultra^®^ glucometer (LifeScan, USA)*.* Then the mice were injected intraperitoneally with glucose (2.5 g/kg body weight). Blood glucose was measured again after 0.5, 1 and 2 h for the IPGTT.

### 3.8. Statistical Analysis

Data are presented as mean ± SD. Statistical analysis of the data for multiple comparisons was performed by analysis if variance (ANOVA). For single comparison, the significance of differences between means was determined by *t*-test. Values of *P* < 0.05 were considered statistically significant, and a value of *P* < 0.001 was considered statistically most significant.

## 4. Conclusions

In summary, we have discovered a novel synthetic analogue of bromophenol HPN and evaluated its anti-diabetic activities in *db*/*db* mice. Both the results of anti-diabetic activities *in vitro* and *in vivo* are inspiring. Hence, the chemical entity reported in this study could provide a possible opportunity for developing a novel PTP1B inhibitor with promising pharmacological properties. The acute toxicity assay of HPN is in progress and will be reported in due course.
